# Sleep-Disordered Breathing in Children With Metabolic Syndrome: The Role of Leptin and Sympathetic Nervous System Activity and the Effect of Continuous Positive Airway Pressure

**DOI:** 10.1542/peds.2008-0154

**Published:** 2008-09

**Authors:** Neal Nakra, Sumit Bhargava, James Dzuira, Sonia Caprio, Alia Bazzy-Asaad

**Affiliations:** a Section of Respiratory Medicine, Yale University School of Medicine, New Haven, Connecticut; b Yale Center for Clinical Investigation, Department of Pediatrics, Yale University School of Medicine, New Haven, Connecticut; c Section of Endocrinology, Yale University School of Medicine, New Haven, Connecticut

**Keywords:** metabolic syndrome, sleep apnea, CPAP, leptin

## Abstract

**Objective:**

The purpose of this work was to determine whether, in children with metabolic syndrome and sleep-disordered breathing, metabolic markers separate them from children with metabolic syndrome without sleep-disordered breathing and whether treatment of sleep-disordered breathing with continuous positive airway pressure is associated with an improvement in metabolic derangement.

**Patients and Methods:**

Subjects aged 7 to 19 years old with metabolic syndrome and a positive validated sleep questionnaire were recruited. Subjects underwent overnight polysomnography, during which sympathetic nervous system activity was assessed via 8-hourly measurements of norepinephrine and epinephrine, together with leptin. The next morning, a fasting 3-hour oral glucose-tolerance test was performed to calculate whole-body insulin sensitivity. A fasting lipid panel interleukin 6, adiponectin, and C-reactive protein levels were also measured. Children with sleep-disordered breathing were placed on continuous positive airway pressure for 3 months and studied again. Sleep-disordered breathing and no sleep-disordered breathing groups were compared by using Fisher's exact test and *t* test for independent samples with analysis of covariance to adjust for age and BMI.

**Results:**

Of 34 children studied, 25 had sleep-disordered breathing (apnea-hypopnea index: >1.5). Mean hourly norepinephrine and leptin levels were higher in the group with sleep-disordered breathing compared with the group without sleep-disordered breathing (P < .005), with no difference in whole-body insulin sensitivity. Eleven subjects with sleep-disordered breathing completed 3 months of nightly continuous positive airway pressure treatment. In the follow-up study, mean hourly leptin levels were significantly lower than in the initial study, with no change in BMI *z* score or other measurements.

**Conclusion:**

Our findings support the hypothesis that sleep-disordered breathing in children with metabolic syndrome is associated with increased sympathetic nervous system activity and leptin levels but not worsening of insulin resistance. Treatment of sleep-disordered breathing with continuous positive airway pressure led to a significant decrease in leptin levels.

As in Adults, obesity in children is associated with a host of problems, including hypertension, dyslipidemia, chronic inflammation, increased blood clotting tendency, endothelial dysfunction, and hyperinsulinemia.^1–7^ In 1988, Reaven^8^ described a significant relationship between obesity and these derangements, coining the term “insulin resistance syndrome” or “syndrome X,” later referred to as metabolic syndrome.^9^ Using modified pediatric criteria to define metabolic syndrome, Weiss et al^10^ reported that, in a group of obese children aged 4 to 20 years old, the prevalence of metabolic syndrome was 38.7% in moderately obese and 49.7% in severely obese children.

Sleep-disordered breathing (SDB) is commonly found in both obese adults and children.^11^ In adults, SDB has long been associated with increased metabolic and cardiovascular morbidity. While awake, adults with SDB have high baseline sympathetic nervous system activity (SNSA), which further increases during sleep, especially in association with apneas and arousals from apneas.^12^ Release of leptin, a hormone with multiple functions, including regulating food intake, has been shown to be inhibited by SNSA.^13^ It would, therefore, be expected that, in individuals with SDB, leptin levels would be low. However, adults with obstructive sleep apnea (OSA) have both high SNSA and high leptin levels, suggesting possible “leptin resistance.”^14^ In 1 study, the diagnosis of OSA in adults, independent of obesity, was associated with a ninefold increase in insulin resistance, type 2 diabetes mellitus, hypertension, and/or dyslipidemia, all components of the metabolic syndrome.^15^ Whether a similar association is seen in obese children is unclear. In addition, the mechanisms by which SDB and metabolic syndrome are linked and whether treatment of one will affect the severity of the other in the pediatric population remain to be elucidated.

In this study, our aim was to determine whether SDB was present in children with metabolic syndrome. We hypothesized that children with metabolic syndrome and SDB have increased SNSA, leptin levels, and insulin resistance and that treating SDB will be associated with improvement in metabolic derangements.

## Methods and Materials

### Study Population

Children aged 7 to 19 years with metabolic syndrome were recruited for this study. Metabolic syndrome was defined as demonstrating ≥3 of the following, on the basis of age, gender, and ethnicity^10^: BMI of ≥97th percentile, triglycerides level ≥95th percentile, high-density lipoprotein (HDL) at <5th percentile, systolic or diastolic blood pressure at ≥95th percentile, and impaired glucose tolerance (glucose between 140 and 200 mg/dL in the oral glucose-tolerance test [OGTT]).

These subjects with metabolic syndrome were recruited from the Yale Pediatric Obesity Clinic and from children who were referred to the Yale Pediatric Sleep Center for evaluation of SDB. All of the subjects were first screened for SDB using a validated pediatric questionnaire^16^ and enrolled if the questionnaire was suggestive of SDB. Informed consent was obtained either at the clinic visit or the afternoon before the polysomnography. The study protocol was approved by the Yale Institutional Review Board.

### Study Design

On the evening of the study, an indwelling catheter was placed for blood sampling. The subject was given dinner before the polysomnography and not fed after 10 pm. During the polysomnography, a 5-mL blood sample was drawn every hour to measure epinephrine and norepinephrine levels and to assess SNSA and leptin levels. The next morning, the subject underwent a 3-hour OGTT. Before the OGTT, fasting levels of interleukin (IL) 6, adiponectin, and C-reactive protein (CRP) were measured, and a lipid panel was run. On the basis of the results of the polysomnography, subjects were identified as either SDB-positive or SDB-negative. The data were compared between these 2 groups.

The diagnosis of SDB was confirmed if, over the first 4 hours of the polysomnography, the subject had an apnea-hypopnea index (AHI) of ≥ 1.5 per hour.^17–19^ Subjects with SDB were then placed on continuous positive airway pressure (CPAP) during the second half of the study, and the pressure was titrated until the AHI was < 1 per hour. Subjects were instructed to use this level of CPAP while asleep at home every night for the next 3 months. Compliance with CPAP treatment was monitored by 1 of the investigators (N.K.N.), who called each subject every 2 to 4 weeks and inquired about CPAP use. The home CPAP machine (S8 elite [ResMed, Poway, CA]) contained a data card that detailed the actual amount of CPAP usage, measuring the number of hours each night that a vacuum seal was made with the mask. The downloaded data from the data card were e-mailed to the investigator.

### Procedures

The study was performed in a darkened room with the subject sleeping in a comfortable bed. A parent was allowed to stay with the child overnight. Standard testing consisted of recording the electroencephalogram (C3/A2 and C4/A1), electromyogram (chin and tibia), electrooculogram (right and left), electrocardiogram, pulse oxygen saturation (SPo_2_), nasal end-tidal CO_2_, nasal-oral airflow by thermistor, and qualitative thoracic/abdominal movement by respiratory-inductive plethysmography (RIP). Subjects with evidence of SDB during the first half of the night were placed on CPAP for the second half of the night, and pressure was gradually titrated to a level that relieved the airway obstruction, as evidenced by elimination of snoring, respiratory effort, and hypoxemia and a reduction of AHI to < 1 per hour. Three subjects required an additional night for complete CPAP titration.

Sleep was staged using the criteria of Kales and Rechtschaffen.^20^ Arousals were scored according to the standards of the American Sleep Disorders Association.^21^ Respiratory variables were scored according to standards established by the American Thoracic Society and previously published data on children.^21,22^ Obstructive apnea was defined as absence of airflow for ≥2 respiratory cycles, associated with paradoxical movement of the chest and abdomen. Hypopneas were defined as a decrease of ≥50% in oronasal thermistor signal and a concurrent arousal and/or fall of ≥3% in SPo_2_ from baseline. Central apneas were defined as the absence of both airflow and respiratory effort (detected by RIP) that was not immediately preceded by an arousal or awakening and that lasted for ≥20 seconds and/or events <20 seconds associated with a fall of ≥3% in SPo_2_ from baseline. An index composed of the number of events (central apnea, obstructive apnea, and obstructive hypopnea) per hour of sleep was then calculated. An AHI of ≥1.5 per hour signified a positive polysomnography result and, hence, the diagnosis of SDB.^17–19^ Mixed apneic events were counted as obstructive. The appearance of paradoxical breathing was determined qualitatively by the asynchronous appearance of the chest wall and abdominal tracing.

### Biochemical Analyses

The OGTT was performed as reported previously.^23^ Glucose and insulin were drawn every 30 minutes for a total of 3 hours to calculate the whole-body insulin-sensitivity index.

Plasma adiponectin, insulin, and leptin levels were measured using a double antibody radioimmunoassay (Millipore, Billerica, MA). Norepinephrine and epinephrine levels were measured using high-performance liquid chromatography with electrochemical detection after extraction (ESA, Chelmsford, MA). CRP levels were measured with the King Diagnostics high-sensitivity CRP assay (Kings Diagnostics, Vista, CA; intra-assay coefficient of variation: 2%; interassay coefficient of variation: 4%). Plasma glucose levels were measured with the YSI analyzer (Yellow Springs Instruments, Yellow Springs, OH), and lipid levels were measured with ACE chemistry analyzer (Alfa Wassermann, West Caldwell, NJ). IL-6 levels were measured using enzyme-linked immunosorbent assay (R&D Systems, Minneapolis, MN). All of the measurements were performed in the core laboratory of the Yale Center for Clinical Investigation.

### Statistical Analysis

Data are expressed as frequencies (percentages) or means ± SDs as appropriate. SBD and non-SBD groups were compared by using Fisher's exact test and *t* test for independent samples. Analysis of covariance was performed to compare groups after adjustment for age and BMI *z* score, whereas paired *t* tests were used to evaluate CPAP treatment. Variability in group differences was quantified by estimating 95% confidence limits.

## Results

**Figure 1:**
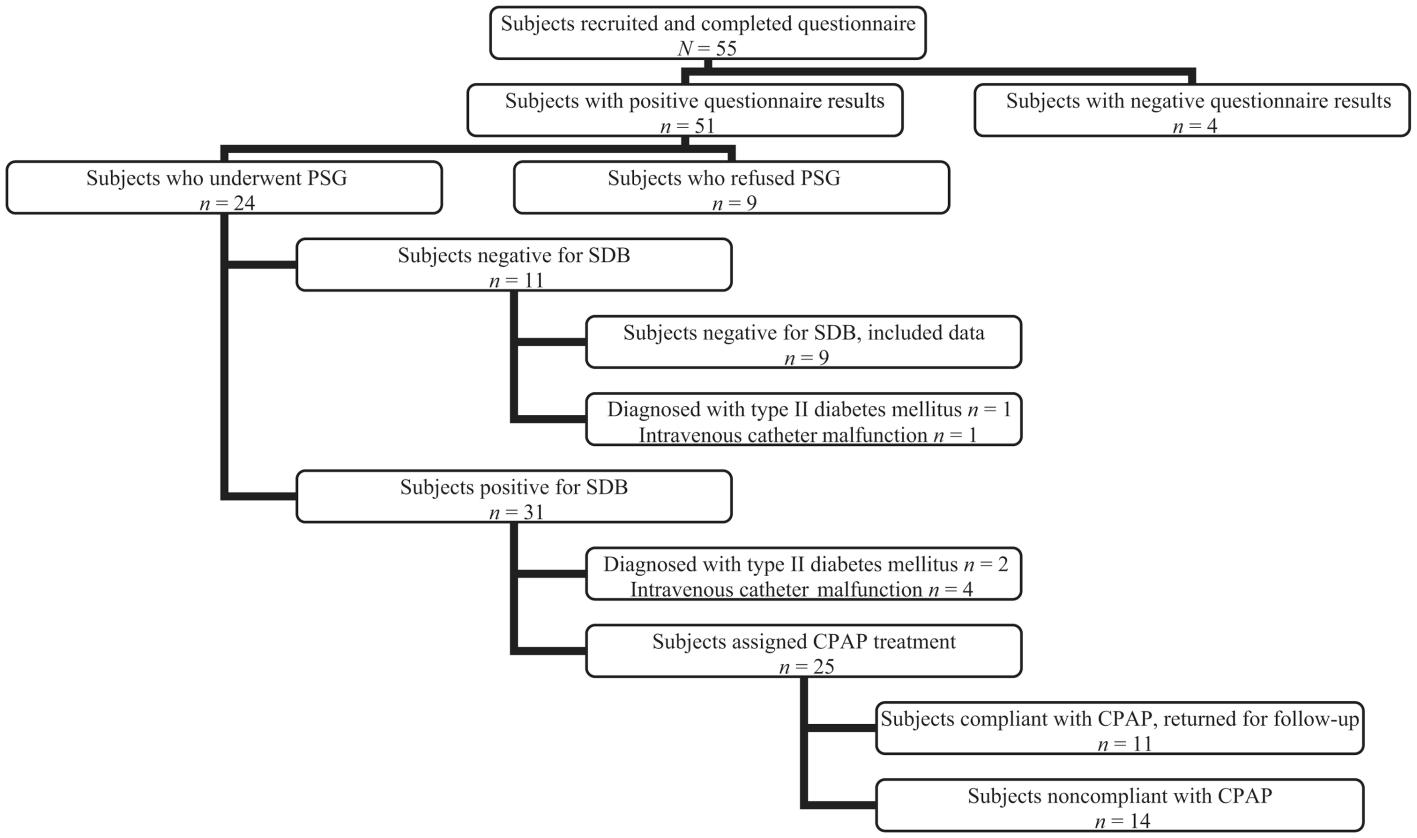
Flow diagram for patient enrollment and follow-up.

Forty-two subjects with metabolic syndrome were enrolled in the study after completing the validated pediatric sleep questionnaire. SDB was confirmed by polysomnography in 31 of the 42 subjects. Eight subjects (6 with SDB and 2 without SDB) were excluded from final data analysis for various reasons (Fig 1). Previously undiagnosed type 2 diabetes was identified in 3 subjects (2 with SDB and 1 without SDB) the morning after the polysomnography on the basis of the results of the OGTT, which disqualified these children from the study.

Of the remaining 34 subjects who were included in the data analysis, 25 subjects (74%) had SDB. Because excessive daytime sleepiness or hyperactivity may occur in association with SDB, we analyzed the number of positive responses to the 4 questions in the screening questionnaire that addressed excessive daytime sleepiness and hyperactivity. In the SDB group, the mean number of positive responses was 2.6, as compared with 2.5 in the non-SDB group (*P* > .1). The male/female ratio was comparable in subjects with and without SDB.

Among all 34 of the subjects, 11 (32%) were 7 to 10 years old, 16 (61%) were between 11 and 17 years old, and only 2 subjects (6%) were 18 to 19 years old. There was no correlation between age and AHI or between BMI and AHI. The ethnic composition of the subjects included 11 black subjects (32%), 6 white subjects (18%), and 17 Hispanic subjects (50%). The proportion of black and white subjects tended to be higher in the SDB group; however, this difference was not significant (*P* = .17) and was consistent with previous reports of increased risk of SDB in black children and adolescents.^24–26^

As shown in Table 1, subjects in the 2 groups were of comparable age, BMI (≥95th percentile for age and gender), and BMI *z* score. Only 1 of the 9 subjects without SDB, compared with 10 of 25 subjects with SDB, had a history of a previous adenotonsillectomy (*P* = .21). By definition, the SDB group had a significantly higher mean AHI as compared with the group without SDB. Except for total cholesterol, there was no difference in the lipid profile between the 2 groups (Table 1).

### SNSA, Leptin, and Insulin Sensitivity in the Presence of SDB

**Figure 2:**
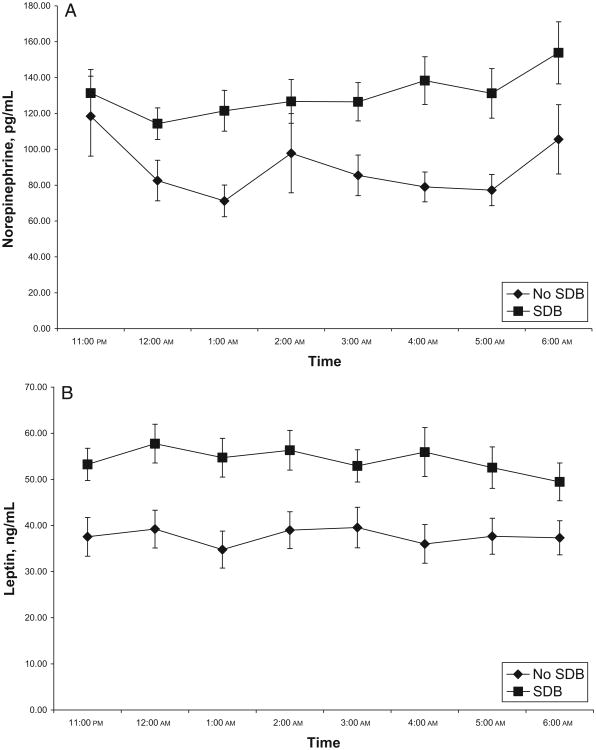
Mean (± SE) hourly norepinephrine (A) and leptin (B) levels for SDB and non-SDB groups.

The mean hourly norepinephrine level, but not epinephrine, was significantly higher in subjects with SDB, as was the mean hourly leptin level (Table 2). The same difference was seen in the profile of norepinephrine and leptin over the 8-hour study period (Fig 2). In contrast, insulin sensitivity (whole-body insulin-sensitivity index) was not different between the groups (Table 2). The differences in norepinephrine and leptin levels remained statistically significant after controlling for age and BMI *z* score with analysis of covariance.

### Inflammatory Markers

We compared the anti-inflammatory and proinflammatory markers adiponectin, IL-6, and CRP in the 2 groups, none of which were significantly different between the groups (Table 3).

### Effect of CPAP Treatment

Despite biweekly calls from the principal investigator and the compliance data card that was included with every CPAP machine, only 11 of the 25 SDB subjects completed the CPAP treatment. Ten of the 14 subjects who were nonadherent cited difficulty adjusting to nightly CPAP usage, 2 subjects were lost to follow-up, and 2 additional subjects were unable to procure the CPAP device.

**Figure 3:**
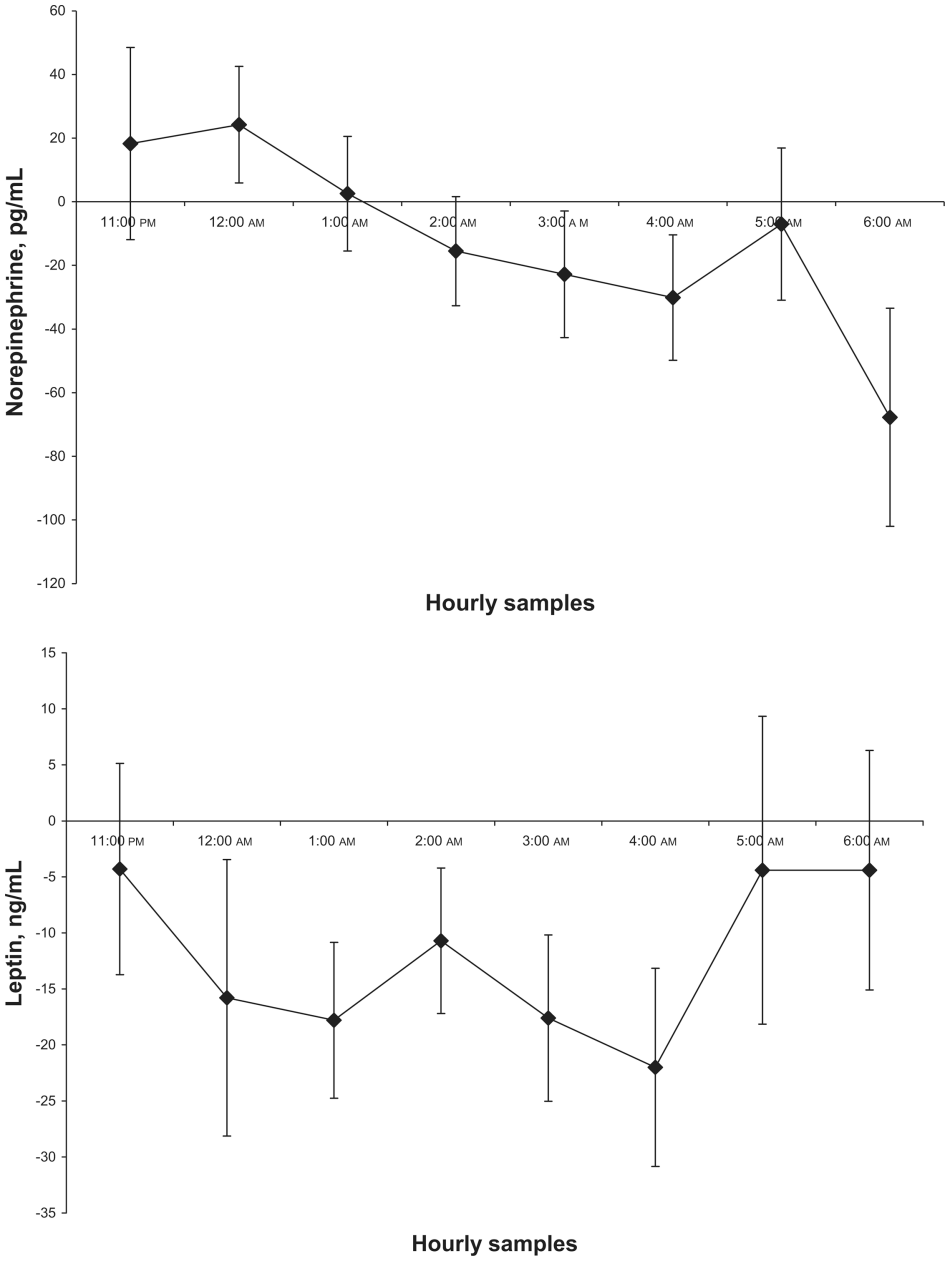
Mean difference between first and second sleep studies in the SDB group.

There was no change in the BMI or BMI *z* score between the initial and follow-up polysomnography for the 11 subjects who did complete the CPAP treatment (Table 4). The mean norepinephrine and leptin levels did decrease, but only the leptin levels decreased significantly (*P* < .031) (Fig 3). Neither the OGTT nor the inflammatory markers were significantly different between the 2 polysomnography studies.

## Discussion

In this study we identified SDB in a group of children with metabolic syndrome in whom SDB had not been suspected previously. In these children, mean norepinephrine and leptin levels were increased compared with the children with metabolic syndrome and no SDB. Treatment of SDB with CPAP was associated with an improvement in nocturnal leptin.

The presence of SDB in the children that we studied was not surprising and is consistent with previous studies that have shown that SDB is prevalent in obese children,^27^ and SDB is underdiagnosed in children.^28^ It was unclear, however, how common SDB was in this group of extremely obese children with metabolic abnormalities. Although the study was not designed to determine actual prevalence, because the number of subjects evaluated was small, we found that the majority of children who were screened for SDB were confirmed by polysomnography to have it. Consistent with other studies,^29–31^ we also found that a proportion of children with SDB had a history of adenotonsillectomy (40%), presumably for OSA, yet residual SDB had not been identified before their recruitment to the study. As in previous studies, our findings stress the importance of follow-up of obese children with SDB and evaluation for residual SDB.

We also demonstrated an increase in SNSA (higher mean hourly norepinephrine levels) in the SDB group compared with the non-SDB group. This finding is consistent with studies of adults with OSA in which investigators assessed SNSA by using a variety of methods.^13,32–35^ Our decision to assess SNSA by sampling serum catecholamines was based on our plan to also measure serum leptin levels throughout the night. Our finding that there was no difference in epinephrine between the groups is consistent with recent studies that show that norepinephrine is a more sensitive marker of SNSA than epinephrine.^36,37^

Because sympathomimetic amines have been shown to inhibit leptin gene expression,^14^ it had been suggested that leptin levels in adults with OSA may be low secondary to the high SNSA, the inherent sleep deprivation that OSA causes, and the fact that leptin elicits a decrease in appetite.^38^ However, as in adults with OSA who have both high SNSA and high leptin levels,^39–41^ we found that this group of children also had both increased SNSA and leptin, as compared with the non-SDB group. Our latter finding of higher leptin levels in the SDB group is consistent with a study by Tauman et al,^42^ who showed that leptin levels correlate directly with BMI *z* score and AHI. In our study, leptin levels were higher in the SDB group, which had comparable BMI *z* scores to the non-SDB group.

In adults, the degree of insulin resistance seems to correlate directly with the severity of both SDB and nocturnal hypoxemia.^43,44^ In a large, population-based study, severe SDB (AHI: >20 per hour) imparted a fivefold risk of overt diabetes mellitus.^45^ In another study of 261 adult patients, an escalation in insulin concentration was seen in obese patients with elevated AHI, with the highest insulin levels present in patients with BMI >29 and AHI >25, suggesting an additive effect of OSA and obesity on insulin resistance.^46^ We hypothesized that, in our subjects with SDB, insulin sensitivity would be decreased and higher levels of inflammatory markers would be found compared with subjects without SDB. However, we found no correlation between SDB and insulin sensitivity in our study. This is consistent with more recent data finding that obesity, rather than SDB, is a major factor in insulin resistance.^47–49^

The lack of a significant difference in insulin sensitivity could also be explained by the fact that our comparison group was morbidly obese children with metabolic syndrome rather than a control group of obese subjects without metabolic syndrome or lean, healthy control subjects. Another possibility is that, because of the small sample size, a difference between the 2 groups was not observed. Finally, it is possible that there is no relationship between SDB and insulin resistance in this age group and particular stage of the syndrome and that an association is seen later with the progression of obesity and its comorbidities.

To further elucidate the proposed relationship among SNSA, leptin, SDB, and insulin sensitivity, we treated SDB with CPAP. Many studies have examined the effect of CPAP treatment of OSA on insulin sensitivity, and among these studies there have been conflicting results.^50–53^ One of our objectives was to determine whether treating SDB with CPAP would have a moderating effect on the metabolic derangements in these children. To our knowledge, this is the first study examining these effects in children with metabolic syndrome.

In children, adenotonsillectomy is currently the first-line approach to treating SDB. However, there were several reasons that we chose CPAP as our treatment modality. SDB associated with obesity may differ from SDB associated with adenotonsillar hypertrophy, and, therefore, adenotonsillectomy as the first-line treatment may not alleviate SDB.^54^ We found this to be the case, because 40% of our subjects had already undergone adenotonsillectomy, and the next line of treatment would be CPAP.^29,55–57^ Finally, the possible confounding variable of weight change secondary to treatment, as observed in other studies,^30,31^ was felt to be best minimized by CPAP for a finite period of time, that is, 3 months.

Although we planned to treat all of the subjects with SDB with CPAP for 3 months, we found that compliance with CPAP was low, with only approximately one third of the subjects completing this phase of the study. This occurred despite a high level of support, with biweekly physician calls and 24-hour availability for any problems concerning the CPAP device. Our rate of return for follow-up polysomnography was lower than in a previous multicenter study in children designed to detect adherence and effectiveness of positive airway pressure.^58^ As a result, we had a small number of subjects for data analysis, which may explain the lack of a difference between the pre-CPAP and post-CPAP biochemical data. Of note, there was a trend toward improvement in the mean fasting glucose, insulin, and insulin-sensitivity levels, as well as a decrease in mean IL-6 and CRP, none of which, however, were statistically significant.

## Conclusions

We demonstrate that, in this group of obese children with metabolic syndrome, previously undiagnosed SDB was associated with increased SNSA and higher leptin levels and that treating SDB with CPAP was associated with a decrease in leptin levels. The implications of our findings are that it may be possible to have an impact on the metabolic derangements in this population if SDB is diagnosed and treated. Future studies with large numbers of subjects and control groups that would include obese and lean children without metabolic syndrome are, therefore, needed.

## Figures and Tables

**Table 1:** Demographics and Baseline Lipid Profile of Subjects With and Without SDB

Variable	SDB (*N* = 25)	No SDB (*N* = 9)	*P*
Gender, male, female	12, 13	5, 4	>.1
Male, female, %	52, 48	56, 44	>.01
Ethnicity, *n* (%)			
Black	10 (91)	1 (9)	.007
White	5 (83)	1 (17)	.102
Hispanic	10 (58)	7 (42)	.467
Age, mean ± SD, y	13.1 ± 3.0	12.3 ± 3.4	.55
BMI, mean ± SD, *z* score	2.8 ± 0.26	2.57 ± 0.29	.66
History of T&A, *n* (%)	10 (40)	1 (11)	.07
AHI per h, mean ± SD	8.68 ± 9.63 (range: 2–48)	0.97 ± 0.44	.001
Total cholesterol, mean ± SD, mg/dL	152.0 ± 29.0	181.3 ± 34.6	.042
HDL, mean ± SD, mg/dL	38.9 ± 9.9	34.4 ±5.8	.12
LDL, mean ± SD, mg/dL	91.8 ± 23.7	108.3 ± 29.8	.16
Triglycerides, mean ± SD, mg/dL	106.8 ± 67.3	205.1 ± 124.6	.05

T&A indicates adenotonsillectomy; LDL, low-density lipoprotein.

**Table 2:** Mean Catecholamine, Leptin, and Fasting Glucose Levels and Insulin-Sensitivity Index in Subjects With And Without SDB

Variable	SDB (*n* = 25), Mean ± SD	No SDB (*n* = 9), Mean ± SD	*P*	95% Confidence Limits	

				Lower	Upper
Norepinephrine, pg/mL	130.9 ± 42.1 (*n* = 24)	89.7 ± 27.3	.003	−67.2	−15.3
Epinephrine, ng/mL	13.4 ± 18.1 (*n* = 24)	7.3 ± 3.0	.13	−13.9	1.9
Leptin, ng/mL	54.2 ± 18.9 (*n* = 24)	37.6 ±11.0	.005	−30.3	−2.8
Fasting glucose, mg/dL	100.7 ± 11.4	103 ± 11.5 (*n* = 8)	.54	−6.8	12.3
Whole-body insulin-sensitivity index	1.45 ± 0.89	1.14 ± 0.47 (*n* = 8)	.22	−0.8	0.2

**Table 3:** Inflammatory Markers in Subjects With And Without SDB

Variable	SDB (*n* = 25), Mean ± SD	No SDB (*n* = 9), Mean ± SD	*P*
Adiponectin, mg/mL	8.3 ± 3.8	7.9 ± 3.3 (*n* = 8)	.78
IL-6, pg/mL	3.9 ± 4.3 (*n* = 24)	3.0 ± 2.4	.24
CRP, mg/dL	0.49 ± 0.43	0.48 ± 0.84	.74

**Table 4:** Difference Before and After CPAP Treatment of the SDB Group

Variable	Mean Difference ± SD	*P*	95% Confidence Limits	

			Lower	Upper
Age, mo	4.910 ± 2.30	NA	0.28	0.54
BMI *z* score	−0.028 ± .090	.33	−0.09	0.03
Norepinephrine, pg/mL	−5.800 ± 45.200	.7	−38.1	26.5
Leptin, ng/mL	−8.350 ± 10.400	.031	−15.8	−0.94
Whole-body insulin-sensitivity index	0.110 ± 0.560	.6	−0.29	0.50

*P* value is based on 1 sample *t* test. NA indicates not applicable.

## What's Known on This Subject

SDB is observed in obese children and adults. Whether SDB is common in children with metabolic syndrome and metabolic markers separate the children with metabolic syndrome and SDB from those without SDB is unclear.

## What This Study Adds

Diagnosing and treating SDB in children with metabolic syndrome may lead to an improvement in some of the metabolic abnormalities associated with metabolic syndrome.

## Abbreviations

SDB: sleep-disordered breathing
SNSA: sympathetic nervous system activity
OSA: obstructive sleep apnea
HDL: high-density lipoprotein
OGTT: oral glucose-tolerance test
IL: interleukin
CRP: C-reactive protein
AHI: apnea-hypopnea index
CPAP: continuous positive airway pressure
SPo_2_: pulse oxygen saturation
